# Toroidal Localized Spoof Plasmons on Compact Metadisks

**DOI:** 10.1002/advs.201700487

**Published:** 2017-12-31

**Authors:** Pengfei Qin, Yihao Yang, Muhyiddeen Yahya Musa, Bin Zheng, Zuojia Wang, Ran Hao, Wenyan Yin, Hongsheng Chen, Erping Li

**Affiliations:** ^1^ Key Laboratory of Micro‐Nano Electronics and Smart System of Zhejiang Province Department of Information Science & Electronic Engineering Zhejiang University Hangzhou 310027 China; ^2^ Zhejiang University‐University of Illinois at Urbana‐Champaign Institute Zhejiang University Haining 314400 China; ^3^ State Key Laboratory of Modern Optical Instrumentation and The Electromagnetics Academy at Zhejiang University Zhejiang University Hangzhou 310027 China; ^4^ School of Information Science and Engineering Shandong University Jinan 250100 China

**Keywords:** localized spoof plasmons, metamaterials, toroidal dipoles

## Abstract

Localized spoof surface plasmons (LSSPs) have recently emerged as a new research frontier due to their unique properties and increasing applications. Despite the importance, most of the current researches only focus on electric/magnetic LSSPs. Very recent research has revealed that toroidal LSSPs, LSSPs modes with multipole toroidal moments, can be achieved at a point defect in a 2D groove metal array. However, this metamaterial shows the limitations of large volume and poor compatibility to photonic integrated circuits. To overcome the above challenges, here it is proposed and experimentally demonstrated compact planar metadisks based on split ring resonators to support the toroidal LSSPs at microwave frequencies. Additionally, it is experimentally demonstrated that the toroidal LSSPs resonance is very sensitive to the structure changes and the background medium. These might facilitate its utilization in the design and application of plasmonic deformation sensors and the refractive index sensors.

Surface plasmon polaritons (SPPs) are a kind of electromagnetic (EM) surface wave propagating at the interface between noble metals and dielectrics at optical frequencies.^1^, ^2^, ^3^, ^4^, ^5^ With the ability to confine EM waves at a deep subwavelength scale, SPPs provide solutions to overcome the diffraction limit and miniaturize the photonic components,^6^ which is promising for various applications, such as miniaturized sensors,^7^ photonic circuits,^8^, ^9^ light localization,^10^, ^11^ photovoltaics,^12^ etc. Extending the concept of surface plasmon polaritons to longer wavelength regimes, such as microwave and terahertz frequencies, will be of great benefit to miniaturize the components of integrated circuits.^1^ However, at these frequency regions the noble metals behave like perfect electric conductors (PECs) and hence can only support surface wave with weak confinement. To increase the confinement, a concept of spoof SPPs has been proposed by Pendry et al. in 2004, where a special surface wave mode, propagating on a periodically subwavelength structured PEC surface, has enhanced energy confinement and a dispersion similar with that of natural SPPs but at much lower frequencies.^13^ Various methods have been developed to geometrically design the system of spoof SPPs,^14^, ^15^ which lead to potential applications in waveguiding,^16^, ^17^, ^18^, ^19^ sensing,^20^, ^21^ laser beams,^22^ etc.

Depending on the geometry, the spoof surface plasmons can either be propagating spoof SPPs or localized spoof surface plasmons (LSSPs).^23^, ^24^ Currently, most of the studies focus on electric/magnetic LSSPs, where the LSSPs show multipole electric/magnetic dipole moments.^25^, ^26^, ^27^, ^28^, ^29^ A toroidal multipole is another form of multipole which cannot be explained by the standard multimode expansion.^30^ It is produced when currents flow on the surface of a torus along its meridian^31^ and has great capability to exist in free space and interacts with light. Because of its unusual EM properties, the toroidal multipoles have attracted increasing attentions recently.^32^, ^33^, ^34^, ^35^, ^36^, ^37^ For example, it has been shown that the dynamic nonradiating charge‐current configurations could produce oscillating and propagating vector potential without emitting EM radiation, which is known as the “anapole moment.”^32^, ^33^, ^34^, ^35^ Despite its physical significance to the light–matter interaction, the research and experimental demonstration of the LSSPs with toroidal dipole moments is rare. Only recently, Kim et al. experimentally demonstrated toroidal LSSPs at a point defect in a 2D groove metal array.^38^ Despite the significance of the work, the large device volume of their structure makes the practical application challenging and difficult to integrate with photonic circuits.

In this work, to overcome the above challenge, we design compact planar metadisks based on split ring resonators (SRRs) to support toroidal LSSPs at microwave frequencies. We successfully observe the near‐field distributions of the toroidal LSSPs resonance mode, both in simulated and experimental results. We study the properties of the toroidal LSSPs and find it very sensitive to the structural parameters and the background medium. This might lead to potential application in plasmonic sensing and integration with the photonic integrated circuits.

In order to design the toroidal LSSPs, we first consider a chain of SRRs as shown in **Figure** **1**a. A single unit cell is depicted at the upper inset of Figure 1a, with a period of *p* = 5.44 mm. The brown region is the SRRs (copper with a conductivity of 5.7 × 10^7^ S m^−1^) where *a* = 9 mm, *s* = *w* = 1.2 mm, *t* = 0.025 mm, and *r_2_* = 0.8 mm. The gray region is the FR4 substrate with a relative permittivity of 3.7 + 0.001 *i* at the frequency below 10.0 GHz. The height and width of the FR4 substrate is *d* = 4 mm, and *l* = 14 mm, respectively. It is well known that the single SRR can produce a magnetic dipole.^39^ By arranging the SRRs in a chain, the induced magnetic dipoles of each SRRs will couple with the SRRs one by one, and in this way the spoof SPPs will be produced. The left inset of Figure 1c depicts this at the frequency of 4.16 GHz, and the direction of the energy flow is represented by the red arrows. The surface current flows in a circle and produces a magnetic dipole along the *x* direction as shown in the right inset of Figure 1c. With these analogies, the respective magnetic dipole moment of SRRs can be shaped according to their arrangement. We may produce the toroidal LSSPs by shaping the chain of the SRRs in a circle, see Figure 1b. The structure consists of 12 pieces of SRRs arranged in a circle on a FR4 substrate. The radius of the substrate is *R* = 20 mm and the distance between the SRRs and the center of the substrate is *r_1_* = 6 mm.

**Figure 1:**
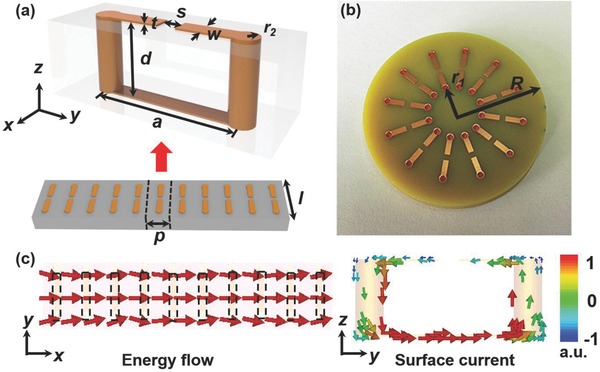
Schematics of the SRR disks. a) A SRR chain. The period of SRRs is *p* = 5.44 mm. The gray region is the FR4 substrate with *d* = 4 mm, *l* = 14 mm, and a relative permittivity of 3.7 + 0.001*i* at 10 GHz. The brown region is the SRRs (copper with a conductivity of 5.7 × 10^7^ S m^−1^) with *a* = 9 mm, *s* = *w* = 1.2 mm, *t* = 0.035 mm, and *r_2_* = 0.8 mm. b) Top view of the proposed structure consisting of 12 SRRs. The radius of the substrate is *R* = 20 mm and *r_1_* = 6 mm. c) Energy flow in the SRR chain and surface current distributions on one of the SRRs at 4.16 GHz. The black dash wireframes represent the SRRs.

To calculate the localized EM modes in the SRR disks, the eigenmode solver of the commercially available software, Computer Simulation Technology (CST) Microwave Studio is employed. **Figure** **2**a,b shows the magnetic and electric field intensity distributions of the SRR disks. The direction of the fields is represented by the black arrows in the figures, where both magnetic and electric fields are strongly confined in a ring‐like form at 4.16 GHz. Moreover, the magnetic field distributions exhibit a vortex with the field that threads all the SRRs, which is produced by the surface current oscillating on the SRRs. Also, the magnetic field is in a shape of torus, which is an important signature of the toroidal dipole moment as it provides a toroidal dipole along *z* direction.

**Figure 2:**
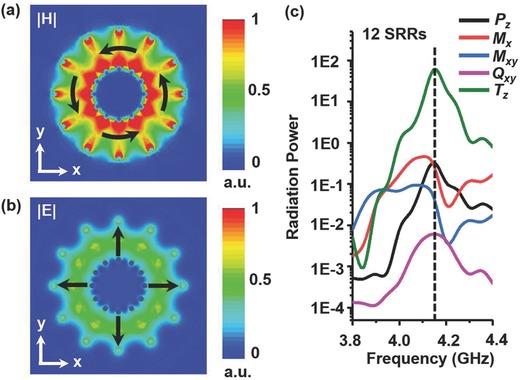
Magnetic and electric field distributions and radiation powers of multiple moments. a) Simulated magnetic and b) electric field intensity distributions in the *xy* plane 2 mm above the SRR disks at 4.16 GHz. The black arrows denote the polarizations of the fields. c) Calculated dispersion of radiation powers of various multipole moments for 12 SRRs. The dash line represents 4.16 GHz.

To fully understand the physics behind this, we investigate the multipole responses in the SRR disks. The modes in the SRR disks are excited by putting a discrete port at the gap of one SRR, and the simulation is performed in the time‐domain solver of CST. With the extracted surface current around 4.16 GHz, the far‐field scattering power of electric dipole, magnetic dipole, toroidal dipole, electric quadrupole, and magnetic quadrupole are calculated according to multipole scattering theory.^40^ The details of the calculations are shown in the Supporting Information. The radiation power magnitude of theses multipoles can be described as follows

(1) $$\begin{array}{c} I\;\;\text{=}\;\;\frac{2\omega^{4}}{3c^{3}}{\left|\vec{P}\right|}^{2}\;\;+\;\;\frac{2\omega^{4}}{3c^{3}}{\left|\vec{M}\right|}^{2}\;\;+\;\;\frac{4\omega^{5}}{3c^{4}}\left|\vec{P}\cdot \vec{T}\right|\;\;+\;\;\frac{2\omega^{6}}{3c^{5}}{\left|\vec{T}\right|}^{2}\;\;+\\ \;\;\;\;\;\frac{\omega^{6}}{5c^{5}}Q_{\alpha \beta }Q_{\alpha \beta }\;\;+\;\;\frac{\omega^{6}}{40c^{5}}\;M_{\alpha \beta }M_{\alpha \beta }\;\;+\;\;o\left(\frac{1}{c^{5}}\right) \end{array}$$

where *c* is the speed of light in the free space, ω is the angular frequency, α, β = *x*, *y* axes in our case, and $\vec{P}$, $\vec{M}$, $\vec{T}$, *Q*_*αβ*_, and *M*_*αβ*_ are the dipole moments of electric dipole, magnetic dipole, toroidal dipole, electric quadrupole, and magnetic quadrupole, respectively. The radiation powers as a function of the frequency are plotted in Figure 2c, where one can see that the toroidal dipole moment is larger than the other dipoles and multipolar components from 3.9 to 4.4 GHz. Especially, the radiation power of toroidal dipole moment is approximately two orders stronger than those of other multipole moments at 4.16 GHz. It is shown that the toroidal dipole moment is dominant around 4.16 GHz and the other resonances are suppressed by the toroidal dipolar resonance.

The variation of the radiation powers with different numbers of SRRs is an important property for the design consideration of the toroidal LSSPs. We calculate the radiation powers of different numbers of SRRs as a function of frequency. **Figure** **3**a,b shows the dispersion of radiation powers of multipole moments in the SRR disks with 8 and 4 SRRs, respectively. It is clear that for the SRR disks with 8 SRRs, the toroidal dipole moment is dominant around 4.14 GHz, while for the SRR disks with 4 SRRs, the toroidal resonance shifts to 4.07 GHz. Figure 3c shows all the radiation powers of the toroidal dipole and resonance frequency in different numbers of SRRs from 4 to 20. One can see that the toroidal resonance frequency blueshifts first and then redshifts when increasing the number of SRRs. This is because when increasing the numbers of SRRs from 4 to 12, the distance between two neighbor SRRs becomes small, the equivalent capacitance *C* slowly decreases due to the weaker edge effect,^41^ while the equivalent inductance *L* almost stays the same when *p* > *d*.^42^ As

(2) $$f\;\;=\;\;\frac{1}{2\pi \sqrt{LC}}$$

the resonance frequency increases. However, when increasing the number of SRRs from 12 to 20, two neighbor SRRs are very close, i.e., *p*<<*d*, the equivalent inductance *L* dramatically increases due to the strong coupling effect.^41^ Though the equivalent capacitance *C* still decreases, the equivalent inductance *L* plays the dominant role and the resonance frequency becomes lower. Note that the radiation power is monotonically increasing as the number of SRRs increases, this is because when number of SRRs increases, confinement of fields is stronger around SRRs, resulting in higher radiation power. To understand the properties of toroidal LSSPs mode, we evaluate the modal volumes and *Q* factors with the formulas

(3) $$V_{m}\;\;=\;\;\frac{\int \varepsilon {\left|E\right|}^{2}dV}{\max(\varepsilon {\left|E\right|}^{2})}$$

(4) $$Q\;\;=\;\;\frac{\omega_{0}}{\Delta \omega }$$

where ε is the dielectric constant, *E* is the electric field, ω_0_ is resonance frequency, and Δω is 3‐dB resonant linewidth.^43^ Figure 3d shows the calculated modal volumes and *Q* factors for different numbers of SRRs. The modal volume decreases and the *Q* factor increases as the number of SRRs increases from 4 to 12. However, both the modal volume and *Q* factor become insensitive to the number of SRRs when the number is larger than 12. This is because when two neighbor SRRs are very close; the field confinement is quite strong, which has little change when continually increasing the number of SRRs.

**Figure 3:**
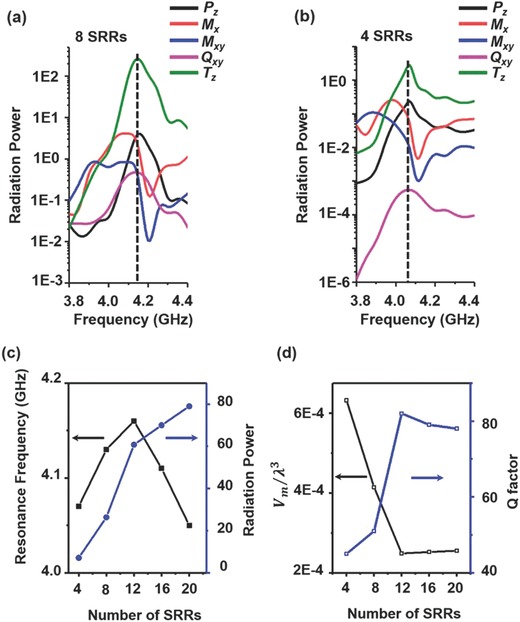
Radiation powers, modal volumes, and *Q* factors of different numbers of SRRs. a) Calculated dispersions of radiation powers of various multipole moments in the SRR disks with 8 SRRs and b) 4 SRRs. The dash line represents 4.14 and 4.07 GHz, respectively. c) Calculated dispersions of radiation powers of toroidal dipole moment in the SRR disks and resonance frequency with 4, 8, 12, 16, and 20 SRRs, respectively. d) Calculated modal volumes and *Q* factors for different numbers of SRRs.

To experimentally verify the toroidal LSSPs mode, we fabricate a sample (Figure 1b) and setup an experiment. In the experimental setup shown in the inset of **Figure** **4**a, a dipole antenna is placed at the gap of one SRR of the SRR disks to excite the LSSPs modes. The discrete port is oscillated along the radial direction of the cylinder substrate. For the detection, another dipole antenna is placed 1 mm above the gap of the SRR to detect the electric field. Both antennas are connected to the vector network analyzer to get the reflection and transmission parameters. From Figure 4a, one can see that there is a dip or a resonance near 4.16 GHz in both simulated and experimental results. This can be explained by the coupled‐mode theory (please see the Supporting Information).^44^ Moreover, around 4.16 GHz the transmission curves experience resonances which are in accordance with our expectation as shown in Figure 4b. All the compared simulated scattering parameters agree well with the experimental demonstration.

**Figure 4:**
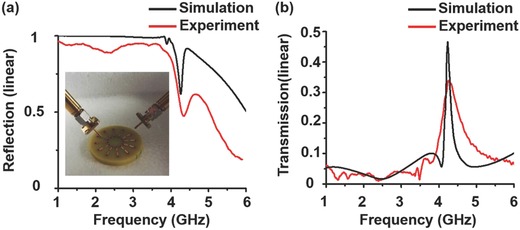
Reflection and transmission parameters. a, b) Simulated and experimental reflection and transmission parameters, respectively.

To scan near field around the metadisks, the detecting dipole antenna is fixed at the arm of a 3D movement platform. It is polarized along the *z* direction 2 mm above the SRR disks. The *E_z_* field distribution measured within 120 mm by 120 mm area experienced the toroidal LSSPs resonance at 4.17 GHz as shown in **Figure** **5**b. The bright spots at the top side of the electric field distributions come from the excitation source. As a comparison, we simulate *E_z_* field distribution of the same setup at 4.17 GHz, see Figure 5a. The experimental and the simulated results show remarkable agreement. From Figure 5a,b, the black dashed lines show the edge of the SRR disks. From the field distributions, one can clearly see that the circulating *E_z_* field varies between negative and positive, which is a dipole‐like EM field radiation pattern. Besides, the location of the dipole is exactly at the center of the SRR disks, which is an important signature of the toroidal LSSPs mode.

**Figure 5:**
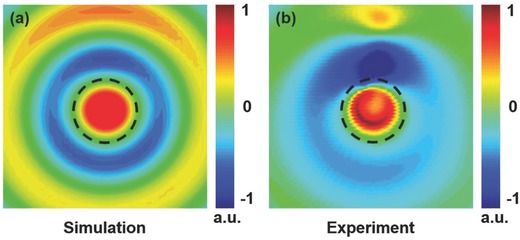
Electric field distributions. a,b) Simulated and experimental *E_z_* electric field distributions in the *xy* plane 2 mm above the structure at 4.17 GHz, respectively. The dipole port is put at the gap of one SRR to excite the toroidal dipole. The black dashed circles represent the locations of the SRR disks.

We also study the impact of the structure and background parameters to the proposed toroidal LSSPs resonator. **Figure** **6**a shows the simulated reflection parameters of toroidal resonator as a function of thickness of the substrate. A redshift of the toroidal LSSPs resonance is observed with increasing thickness of the substrate. For example, when the thickness of the substrate is *t* = 3 mm, the toroidal LSSPs resonance first appears at the frequency of 4.9 GHz. Increasing the thickness of the substrate from 3.5, 4, 4.5, and 5 mm, the toroidal LSSPs resonance is redshifted by 0.35, 0.65, 0.9, and 1.1 GHz, respectively, as shown in Figure 6b. Moreover, Figure 6c shows the simulated reflection parameters as a function of length of the SRRs. A redshift of the toroidal LSSP resonance is observed when increasing the length of SRRs (Figure 6d). Due to the sensitivity of the structure changes, the toroidal LSSPs resonator may be used as a deformation sensor. Similarly, changing the permittivity of the background medium is simulated and demonstrated experimentally. In the simulation, one can see a significant redshift at the resonance frequency when relative permittivity of the background increases, see Figure 6e,f. A similar phenomenon is also experimentally demonstrated, by changing the background medium from air to oil (relative permittivity of 2.5), as shown in Figure 6f. The resonance frequency shifts from 4.17 GHz in the air to 3.7 GHz in the oil and the corresponding resonance frequency shift per refractive index^45^ is 0.8 GHz/refractive index unit. Therefore, the toroidal LSSPs resonator can work as a deformation sensor and to detect the refractive index changes of the surrounding medium.

**Figure 6:**
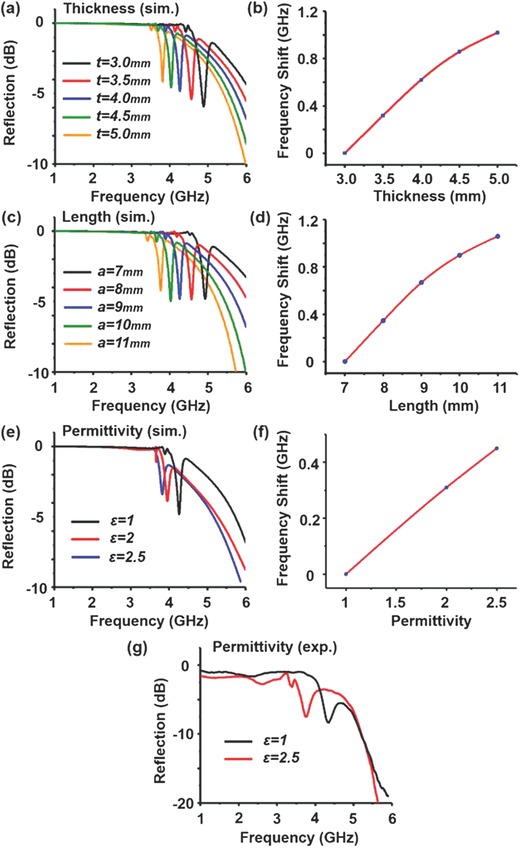
Spectral shifts of toroidal LSSPs resonance. a) Simulated results of reflection parameters with different thickness of the substrate. b) Frequency shift versus thickness of the substrate. c) Simulated results of reflection parameters with different length of the SRRs. d) Frequency shift versus length of the SRRs. e) Simulated reflection parameters, where the relative permittivity of the background is 1, 2, and 2.5. f) Frequency shift as a function of permittivity of the background. g) Measured reflection parameters, where the background is air and oil with the relative permittivity 1 and 2.5, respectively.

In this work, we propose compact planar metadisks which support toroidal LSSPs at microwave frequency regime with strong compatibility with the photonic integrated circuits. The semianalytical results show that a dominant toroidal dipolar response can be observed from 3.9 to 4.4 GHz. We also perform the series of experiments and compare the transmission/reflection parameters and electric field distributions of the toroidal LSSPs mode with the simulation, which clearly indicates a toroidal dipole resonance around 4.17 GHz. Moreover, we numerically and experimentally demonstrate that the proposed toroidal LSSPs resonator is sensitive to the structure changes and surroundings. Therefore, it can work as a plasmonic sensor to detect the deformation of the structure and the refractive index changes of the surrounding medium. Besides, as the present metadisks show high symmetries, when using far field to excite the localized modes on the metadisks, the metadisks show symmetric responses at the toroidal dipole resonant frequency, making the excitations insensitive to the incident angles. Finally, the proposed compact metadisks can also be achieved at higher frequencies, such as terahertz and infrared frequencies, by properly downscaling of the present structure.

## Conflict of Interest

The authors declare no conflict of interest.

## Supporting information

Supplementary
