# Supplementary material for: Toroidal Localized Spoof Plasmons on Compact Metadisks
Source: Adv Sci (Weinh). 2017 Dec 31;5(3):1700487. doi: 10.1002/advs.201700487 (PMC5867056; doi:10.1002/advs.201700487)
Supplement: Supplementary file 1 — Supplementary [file ADVS-5-1700487-s001.pdf]

Copyright WILEY-VCH Verlag GmbH & Co. KGaA, 69469 Weinheim, Germany, 2017.

ADVANCED SCIENCE
Open Access

Supporting Information

for *Adv. Sci.,* DOI: 10.1002/advs.201700487

Toroidal Localized Spoof Plasmons on Compact Metadisks

Pengfei Qin, Yihao Yang, Muhyiddeen Yahya Musa, Bin Zheng, Zuojia Wang, Ran Hao, Wenyan Yin, Hongsheng Chen,* and Erping Li**

Toroidal localized spoof plasmons on compact meta-disks

Pengfei Qin[1,2,3,#], Yihao Yang[1,3,#,*], Muhyiddeen Yahya Musa[1,3], Bin Zheng[1,3], Zuojia Wang[4], Ran Hao[1], Wenyan Yin[1],Hongsheng Chen[1,3*], Erping Li[1,2*]

[1]Key Laboratory of Micro-Nano Electronics and Smart System of Zhejiang Province, Department of Information Science & Electronic Engineering, Zhejiang University, Hangzhou 310027, China.

[2]Zhejiang University-University of Illinois at Urbana-Champaign Institute, Zhejiang University, Haining 314400, China

[3]State Key Laboratory of Modern Optical Instrumentation, and The Electromagnetics Academy at Zhejiang University, Zhejiang University, Hangzhou 310027, China.

[4]School of Information Science and Engineering, Shandong University, Jinan 250100, China.

[#]Co-first authors.

*To whom correspondence should be addressed. E-mail: （Y. Yang）yangyihaooo@zju.edu.cn; (H. Chen) hansomchen@zju.edu.cn; (E. Li) liep@zju.edu.cn

1. Calculation of radiation power of different multipoles

The moments of electric dipole, magnetic dipole, toroidal dipole, electric quadrupole, and magnetic quadrupole can be calculated according to the multipole scattering theory [S1]:

electric dipole moment:

$$\vec{p}_z = \frac{1}{i\omega}\int d^3r\vec{j}, \tag{1}$$

magnetic dipole moment:

$$\vec{m}_{xy} = \frac{1}{2c}\int d^3r\left(\vec{r}\times\vec{j}\right), \tag{2}$$

toroidal dipole moment:

$$\vec{T}_z = \frac{1}{2c}\int d^3r\left[\left(\vec{r}\cdot\vec{j}\right)r - 2r^2\vec{j}\right], \tag{3}$$

electric quadrupole moment:

$$Q_{\alpha\beta} = \frac{1}{i2\omega}\int d^3r\left[r_\alpha j_\beta + r_\beta j_\alpha - \frac{2}{3}\delta_{\alpha\beta}\left(r\cdot j\right)\right], \tag{4}$$

magnetic quadrupole moment:

$$M_{\alpha\beta} = \frac{1}{3c}\int d^3r\left[\left(\vec{r}\times\vec{j}\right)_\alpha r_\beta + \left(\vec{r}\times\vec{j}\right)_\beta r_\alpha\right], \tag{5}$$

where **r** is distance vector from the origin to point (x, y, z) in a Cartesian coordinate system; **j** is current density at the point r (x, y, z); *c* is the speed of light in the vacuum; and $\alpha,\beta = x, y$. The **r** and **j** are exported from CST software at different frequencies. Then we can get the radiation power of each multipole

$$I_p = \frac{2\omega^4}{3c^3}\left|\vec{p}_z\right|^2, \tag{6}$$

$$I_m = \frac{2\omega^4}{3c^3}\left|\vec{m}_{xy}\right|^2, \tag{7}$$

$$I_T = \frac{2\omega^6}{3c^5}\left|\vec{T}_z\right|^2, \tag{8}$$

$$I_Q = \frac{\omega^6}{5c^5} Q_{\alpha\beta} Q_{\alpha\beta}, \tag{9}$$

$$I_M = \frac{\omega^6}{20c^5} M_{\alpha\beta} M_{\alpha\beta}. \tag{10}$$

2. Coupled-mode theory

Based on coupled-mode theory, the coaxial line-toroidal resonator-coaxial line coupling model is shown in Fig. S1, where the coaxial line 1 is with input/output field amplitudes S_{1+}/S_{1-}, coaxial line 2 is with input/output field amplitudes S_{2+}/S_{2-}, and the toroidal resonator is with field amplitude A and resonance frequency ω_0, which is coupled to the coaxial line 1/ coaxial line 2 with a lifetime τ_1/τ_2.

According to the coupled-mode theory, [S2] the reflection spectrum $R(\omega)$ is

$$R(\omega) = \frac{(\omega-\omega_0)^2 + \left(\frac{1}{\tau_1} - \frac{1}{\tau_2}\right)^2}{(\omega-\omega_0)^2 + \left(\frac{1}{\tau_1} + \frac{1}{\tau_2}\right)^2}. \tag{11}$$

We can see that when $\omega = \omega_0$, $R(\omega_0)$ reaches minimum.

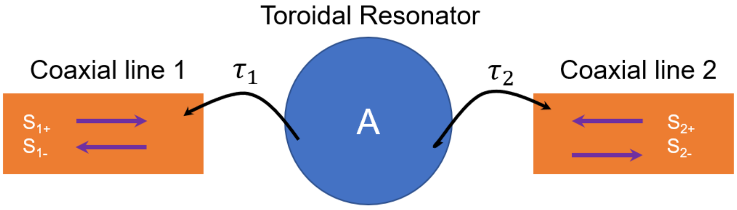

Figure S1. Coaxial line-toroidal resonator-coaxial line coupling model. Here, the coaxial line 1 is with input/output field amplitude S_{1+}/S_{1-}; the coaxial line 2 is with input/output field amplitude S_{2+}/S_{2-}; and the toroidal resonator is with field amplitude A and frequency ω_0 and coupled to coaxial line 1 and 2 with lifetimes τ_1 and τ_2, respectively.

References

[S1]E. E. Radescu, G. Vaman, *Phys. Rev. E* **2002**, *65*, 046609.

[S2]J. D. Joannopoulos, S. G. Johnson, J. N. Winn, R. D. Meade, *Photonic Crystals: Molding the Flow of Light* **2008**, 198.
